# New Rotor Position Redundancy Decoding Method Based on Resolver Decoder

**DOI:** 10.3390/mi13060903

**Published:** 2022-06-07

**Authors:** Anbo Yu, Chenyu Wang, Xiaoqiang Guo, Zheng Li, Chunjiang Zhang, Josep M. Guerrero

**Affiliations:** 1 Department of Electrical Engineering, Yanshan University, Qinhuangdao 066000, China; yuanbo@stumail.ysu.edu.cn (A.Y.); cy.barca@stumail.ysu.edu.cn (C.W.); zhengli@ysu.edu.cn (Z.L.); zcj@ysu.edu.cn (C.Z.); 2 Department of Energy Technology, Aalborg University, 9220 Aalborg, Denmark; joz@et.aau.dk

**Keywords:** resolver, redundancy decode, rotor position, motor

## Abstract

In view of the frequent safety problems of electric vehicles, the research on accurately obtaining the rotor position of the motor through the resolver is an important means to improve the functional safety of the system. The commonly used resolver decoding method involves the resolver decoding chip method and software decoding method, but few studies integrate the two decoding methods. A single method of motor rotor position acquisition cannot meet the requirements of system functional safety. To fill this gap, this paper proposes a method to simultaneously integrate hardware decoding and software decoding in the motor control system. The decoding chip and software decoding obtain the angle data at the same time, and they provide redundancy to improve the functional safety of the electronic control system. Finally, the effectiveness of the proposed simultaneous operation of hardware decoding and software decoding is verified by experiments.

## 1. Introduction

With the advancement of the “carbon peaking and carbon neutrality” target, the new energy vehicle industry has received extensive attention [1]. In response to the national calls, the development of new energy vehicle technology has undoubtedly become a hot topic today [2]. As one of the core technologies of new energy vehicles that is different from traditional vehicles, the motor control system’s safety and accuracy are important indicators to measure the performance of the electronic control system [3]. In view of the current frequent safety problems of electric vehicles, it is very important to study the functional safety of electronic control systems [4].

In the electronic control system, accurately obtaining the rotor position angle of the controlled motor is an important means to improve the functional safety of the system [5]. The main method for obtaining the rotor position angle is the position sensor method and the position sensorless method. The principle of position sensor method is to obtain the rotor position information in real time through the corresponding sensor [6]. Common sensors are Hall sensors, photoelectric encoders, and resolvers. Hall sensors have the disadvantages of low signal level and strict environmental requirements [7], and photoelectric encoders are not suitable for harsh environments, such as high temperature, high humidity, and high dust [8]. Because of its simple structure, high reliability and high precision, the resolver has been widely used in the electric drive system of new energy vehicles [9]. However, the sensorless method is mainly suitable for some constant speed occasions, and its dynamic performance still needs to be improved. In addition, in some occasions where the safety and reliability requirements are very high, and the installation of the sensor is not inherently difficult, the sensor method instead of the sensorless method becomes the best solution. If the two methods are used redundantly in situations with high safety and reliability requirements, due to the difference in accuracy, this may cause the difference between the two to exceed the set threshold and falsely generate a detection fault signal.

As an important part of the electronic control system of new energy vehicles, the resolver can obtain the rotor position angle and speed of the measured motor in real time and accurately, which provides key elements for the control strategy and important feedback information for the maintenance of system security [10].

By judging the amplitude and phase of the output voltage of the rotor winding, the rotor rotating position angle is derived by the resolver and its decoder. Therefore, when using the resolver, the decoding method is crucial to obtain the rotor position angle [11].

At present, the commonly used decoding method in electric vehicles is hardware decoding. However, in recent years, with the development of integrated circuit technology, the software decoding method based on DSP has gradually been applied [12]. The existing resolver-based motor control system has roughly two schemes to obtain the rotor position of the motor. One is to use a resolver decoding chip (such as AD2S1210) to obtain the speed and angle information of the rotor, and the microprocessor of the system reads the result of angle conversion in the decoder chip serially or parallelly; the other is software decoding, that is, the microprocessor of the system sends out a sine excitation wave, and the sine and cosine feedback signal of resolver is connected to AD sampling port of the microprocessor, and the rotor angle information is obtained through a certain algorithm.

In order to improve the accuracy of obtaining the rotor position angle information, most of the literature have optimized the spinning structure. In [13], the proposed resolver structure is optimized to improve the performance of the sensor under mechanical failures. In [14], a novel resolver structure for HEV/EV is proposed. In [15], the resolver winding is improved based on an optimization algorithm. In [16], two configurations of resolver field windings are proposed. There are also studies that were optimized for software decoding. In [17], a method is proposed to generate a sinusoidal digital signal for a state machine. In [18], the quadrature phase-locked loop method is used for the decoder to improve the accuracy. In [19], an estimation algorithm for rotor angular position is proposed. In [20], a digital filter is used to synchronously demodulate the resolver output. However, there are few studies on the integrated use of both soft and hard decoding methods.

For the motor control system, the speed and position information of the rotor are relatively important information. In order to improve the security level of the system, it is necessary to use both a resolver decoding chip and a microprocessor for software decoding in certain situations. The rotor angle and speed information obtained by the user are redundant with each other, so as to obtain the rotor information accurately and reliably.

In this paper, hardware and software decoding are integrated into the motor control system at the same time. The resolver decoding chip is used to send out the excitation voltage, and the decoding chip and software decoding obtain the angle data at the same time. The two decoding methods provide redundancy to improve the functional safety of the electronic control system.

## 2. System Overview

**Figure 1:**
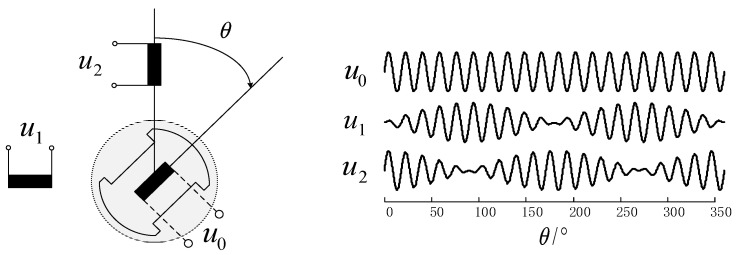
Schematic diagram of resolver structure and voltage waveform.

Figure 1 is a schematic diagram of the traditional resolver structure and the waveform diagram of the feedback voltage changing with the rotor position. When the high frequency sine voltage *u*_0_ is applied on the excitation winding, the feedback sine and cosine voltage (*u*_1_ and *u*_2_) with amplitude modulated by rotor position can be obtained on the feedback winding. The expression is shown in Equation (1), where *ω_ref_* is the angular frequency of the excitation sine voltage, *θ* is the angle of the rotor, and *k* is the turns ratio of the resolver.

(1) $$\begin{array}{l} u_{0}(t)=U_{0}\cdot \sin(\omega_{ref}t)\\ u_{1}(t)=U_{0}\cdot k\cdot \sin(\omega_{ref}t)\cdot \sin\theta \\ u_{2}(t)=U_{0}\cdot k\cdot \sin(\omega_{ref}t)\cdot \cos\theta \end{array}$$

Generally speaking, in order to ensure the normal operation of the resolver decoding chip, it is necessary to use the resolver decoding chip to issue a sine excitation signal. However, this approach will cause a problem for the software decoding module. Since the software decoding module cannot know the exact phase of the excitation voltage, it cannot correctly demodulate the sine and cosine signals, resulting in a large deviation between the results obtained by the software decoding module and the real value. Therefore, it cannot be used as a redundant rotor information acquisition method. This paper gives a corresponding solution to this problem.

In this paper, a method to realize the simultaneous operation of rotary transformer decoding chip and software decoding is presented. The resolver is driven by the excitation voltage generated by the resolver decoding chip, and the rotor information obtained by the two methods is mutually redundant to improve the functional safety level of the system.

When the resolver fails, the two decoding results will be invalid. If the system needs to maintain operation, the rotor information needs to be obtained by the sensorless method. However, this paper only analyzes the two decoding redundancy methods under the resolver.

**Figure 2:**
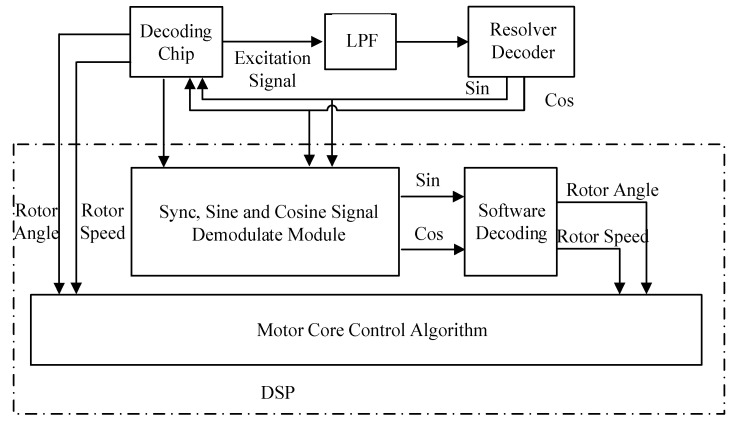
Schematic diagram of the resolver position redundancy decoding scheme.

Figure 2 is the overall block diagram of the system. The circuit required by the original resolver decoding chip is retained in the hardware design. The excitation sine voltage is generated by the resolver decoding chip, and the resolver decoding chip and software decoding run simultaneously. Two methods are proposed below for the “synchronization, sine and cosine signal demodulate module” in the figure.

## 3. The Demodulation Process of Sine and Cosine Signals

The “synchronization, sine and cosine signal demodulate module” in Figure 2 is described in detail, and two schemes are proposed, which are as follows.

### 3.1. Synchronization Module and Sine (Cosine) Signal Demodulation Method 1

**Figure 3:**
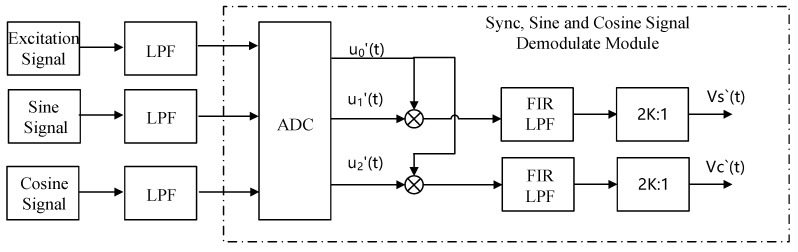
Synchronization, sine and cosine signal demodulation module method 1.

The system inevitably uses low-pass filters in hardware design, and the impact of these filters on the system needs to be considered. The over sampling method is used for the system, and 2K:1 signal extraction is carried out at the end, as shown in Figure 3. The excitation voltage generated by the resolver decoding chip is represented by *u*_0_′(*t*). A phase delay *γ* is introduced in the conditioning circuit for sinusoidal signal extraction, and the excitation signal retrieved by the microprocessor ADC is recorded as *u*_0_′(*t*). The sine and cosine feedback conditioning circuit and the resolver will also introduce a phase delay, which is recorded as *δ*. The turns ratio of the resolver is assumed to be 1, and *θ* is the rotor angle, as shown in Equation (2).

(2) $$\begin{array}{l} u_{0}(t)=U_{0}\cdot \sin(\omega_{ref}t)\\ {u_{0}}^{\prime }(t)=U_{0}\cdot \sin(\omega_{ref}t-\gamma )\\ {u_{1}}^{\prime }(t)=U_{0}\cdot \sin(\omega_{ref}t-\delta )\cdot \sin\theta \\ {u_{2}}^{\prime }(t)=U_{0}\cdot \sin(\omega_{ref}t-\delta )\cdot \cos\theta \end{array}$$

(3) $$\begin{array}{l} V_{S}(t)={u_{1}}^{\prime }(t)\cdot {u_{0}}^{\prime }(t)=\frac{U_{0}^{2}}{2}[\sin\theta \cos(\gamma -\delta )-\sin\theta \cos(2\omega_{ref}-\gamma -\delta )]\\ V_{C}(t)={u_{2}}^{\prime }(t)\cdot {u_{0}}^{\prime }(t)=\frac{U_{0}^{2}}{2}[\cos\theta \cos(\gamma -\delta )-\cos\theta \cos(2\omega_{ref}-\gamma -\delta )] \end{array}$$

(4) $$\begin{array}{l} {V_{S}}^{\prime }(t)=\frac{U_{0}^{2}}{2}\sin\theta \cos(\gamma -\delta )\\ {V_{C}}^{\prime }(t)=\frac{U_{0}^{2}}{2}\cos\theta \cos(\gamma -\delta ) \end{array}$$

*u*_0_′(*t*) is multiplied by *u*_1_′(*t*) and *u*_2_′(*t*), respectively, to obtain *V_S_*(*t*) and *V_C_*(*t*), as shown in Formula (3). In order to improve the sampling accuracy, the oversampling method is used, which is used to sample at a higher frequency, and the oversampling rate is selected as 4. In the later data extraction, 2K = 4 is taken. By FIR low-pass filtering on *V_S_*(*t*) and *V_C_*(*t*), the carrier signal can be filtered out, and only the components related to the rotor information are retained. Finally, data extraction involves resampling at a lower frequency, which improves the signal to noise ratio and amplitude resolution; after 2K:1 data extraction, the demodulated sine and cosine signals *V_S_*′(*t*) and *V_C_*′(*t*) can be obtained, as shown in Equation (4). Here, *V_S_*′(*t*) and *V_C_*′(*t*) correspond to the outputs (sin and cos) of the module in Figure 2, respectively.

It should be noted that in order to ensure the accuracy of the post-stage angle calculation and phase-locked loop, the amplitudes of *V_S_*′(*t*) and *V_C_*′(*t*) should be guaranteed to be near $U_{0}^{2}/2$, that is, the delay angles *γ* is close to *δ*, which need to be properly adjusted when designing the hardware circuit, and the difference should not exceed ±45 degrees.

### 3.2. Synchronization Module and Sine (Cosine) Signal Demodulation Method 2

**Figure 4:**
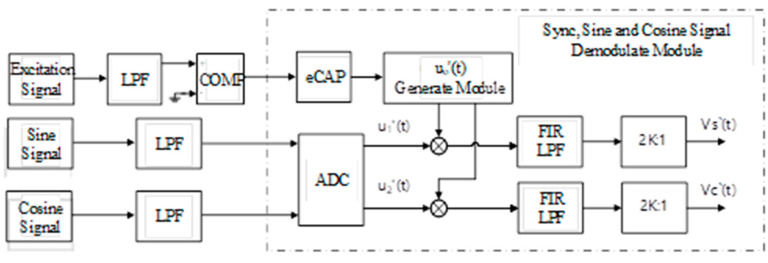
Synchronization, sine and cosine signal demodulation module method 2.

The figure above shows the other demodulation scheme of synchronous, sine and cosine signals. Firstly, the excitation signal is connected to the comparator circuit, and its output is a square wave signal containing the excitation phase and frequency information. The frequency and phase of the excitation signal can be obtained by connecting this signal to the eCAP module of DSP. Therefore, *u*_0_′(*t*) in the Formula (2) can be obtained by means of software, and this process is completed by the “*u*_0_′(*t*) generation module” in Figure 4.

(5) $${u_{0}}^{\prime }(t)=U_{0}\cdot \sin(\omega_{ref}t-\gamma )$$

Different from the above, the delay angle *γ* is adjusted by adjusting the parameters of the hardware circuit and the angle *γ* in the Formula (5) can be obtained by the optimization algorithm in software, so that the amplitudes of the demodulated sine and cosine signals *V_S_*′(*t*) and *V_C_*′(*t*) are near $U_{0}^{2}/2$, which can be obtained from Formula (4), which is as follows:

(6) $$\begin{array}{cc} g & ={\left[{V_{S}}^{\prime }(t)\right]}^{2}+{\left[{V_{C}}^{\prime }(t)\right]}^{2}\\ & ={\left[\frac{U_{0}^{2}}{2}\sin\theta \cos(\gamma -\delta )\right]}^{2}+{\left[\frac{U_{0}^{2}}{2}\cos\theta \cos(\gamma -\delta )\right]}^{2}\\ & =\frac{U_{0}^{4}}{4}\cos{\left(\gamma -\delta \right)}^{2} \end{array}$$

By continuously adjusting the phase of the excitation signal, the value corresponding to the maximum value *g* is the optimal value *γ*. Since the external excitation circuit and the internal sine and cosine demodulation module contain low-pass filters, the phase change speed in the optimization process should not be too fast.

After the *u*_0_′(*t*) signal is obtained, the post-stage processing is the same as the method described above. Finally, the demodulated sine and cosine signals *V_S_*′(*t*) and *V_C_*′(*t*) can be obtained.

After the above demodulation process, the demodulated sine and cosine feedback signals *V_S_*′(*t*) and *V_C_*′(*t*) can be obtained. As shown in Figure 2, the angle and speed of the resolver rotor can be obtained by using the software decoding loop to phase-lock *V_S_*′(*t*) and *V_C_*′(*t*).

## 4. Design of Software Phase Locked Loop

**Figure 5:**
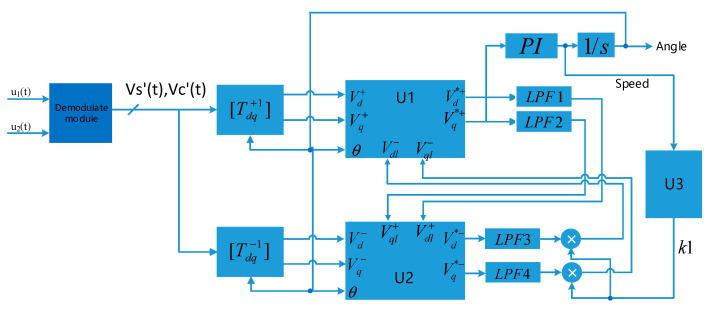
Overall block diagram of software decoding algorithm.

The software decoding algorithm proposed in this paper is shown in Figure 5. *u*_1_′(*t*) and *u*_2_′(*t*) represent the sine and cosine feedback signals of the resolver, respectively, and the demodulated signals *V_S_*′(*t*) and *V_C_*′(*t*) from which the high frequency carrier is removed are obtained through the demodulation module.

**Figure 6:**
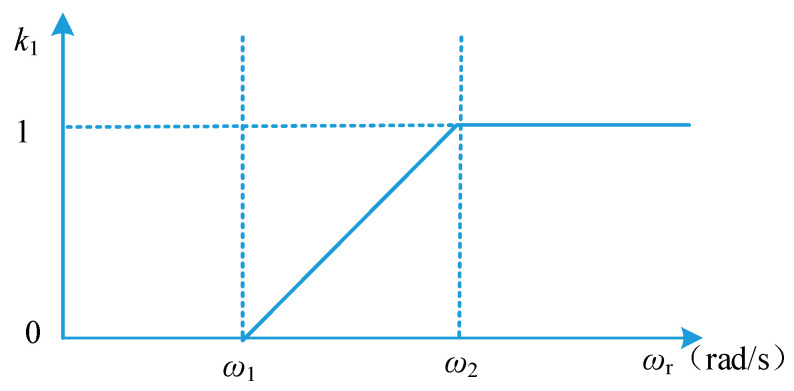
Relationship between weight coefficient k1 and resolver rotor frequency *ω*_r_.

In order to solve the problem of low system bandwidth caused by low cut-off frequency in the low-speed region and static state, the module U3 negative sequence component weight calculation unit is added in Figure 5. According to the magnitude of rotor speed observed by the phase-locked loop, the value of the negative sequence component weight k1 is adjusted in real time, and k1 is multiplied by LPF3 and LPF4, respectively, to obtain the input of the decoupling unit U1. The relationship between the weight coefficient k1 and the resolver rotor frequency *ω*_r_ is shown in Figure 6. According to the value of *ω*_r_, the switching of phase-locked algorithm is realized by adjusting *k*1, so as to improve the rapidity of response. In the low speed region, k1 is 0, and the DDSRF-PLL degenerates into SRF-PLL to achieve a faster resolver rotor position angle phase locking process. In the range of (*ω*_1_, *ω*_2_), k1 linearly transitions from 0 to 1 to achieve smooth phase lock algorithm switching. In the medium and high speed region, the DDSRF-PLL is used to realize the phase locking of the position angle of the resolver rotor. Without loss of generality, it is possible to take $\omega_{1}=2*\pi *30$, $\omega_{2}=2*\pi *50$.

[T_dq_^+1^] and [T_dq_^−1^] in Figure 5 represent the park transformation matrices of the positive sequence coordinate system and the negative sequence coordinate system, respectively. They represent the transformation relationship of components from static coordinate system to forward (reverse) rotating coordinate system. After transformation, the d-q axis voltage components in the positive sequence and negative sequence coordinate systems are obtained, respectively. As shown in Formula (7), they are coupled to each other, where *ε* is an arbitrary quantity, which satisfies the relationship of Equation (7).

(7) $$\begin{array}{l} \left[\begin{array}{c} V_{d}^{+}\\ V_{q}^{+} \end{array}\right]=\left[\begin{array}{l} \begin{array}{cc} \cos(\theta ) & \sin(\theta ) \end{array}\\ \begin{array}{cc} -\sin(\theta ) & \cos(\theta ) \end{array} \end{array}\right]\cdot \left[\begin{array}{c} V_{\alpha }\\ V_{\beta } \end{array}\right]\\ \left[\begin{array}{c} V_{d}^{-}\\ V_{q}^{-} \end{array}\right]=\left[\begin{array}{l} \begin{array}{cc} \cos(\theta ) & -\sin(\theta ) \end{array}\\ \begin{array}{cc} \sin(\theta ) & \cos(\theta ) \end{array} \end{array}\right]\cdot \left[\begin{array}{c} V_{\alpha }\\ V_{\beta } \end{array}\right]\\ \;\left[\begin{array}{c} V_{\alpha }\\ V_{\beta } \end{array}\right]=\left[\begin{array}{c} \cos(\varepsilon )\\ \sin(\varepsilon ) \end{array}\right] \end{array}$$

**Figure 7:**
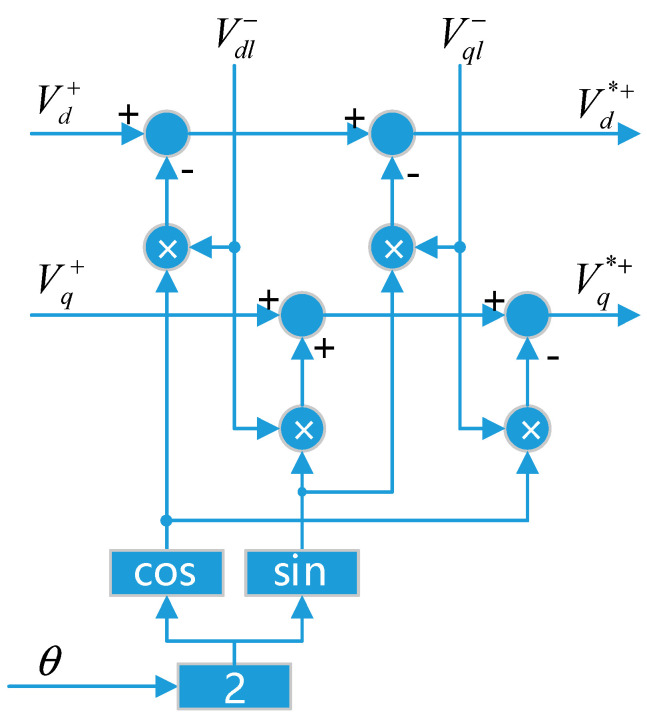
Schematic diagram of decoupling unit (U1).

The decoupling process of the positive and negative sequence components is as follows: the voltage vector [*V_α_*,*V_β_*]*^T^* is composed of two components ${\vec{V}}^{+},{\vec{V}}^{-}$, which are positive and negative sequence components, respectively, which rotate with angular velocities *ω* and −*ω*, respectively, with initial angles $\phi^{+},\phi^{-}$, i.e., *θ* = *ωt*. From this, the mathematical expression shown in Formula (8) can be obtained, and the decoupling unit U1 shown can be obtained. As shown in Figure 7, the decoupling unit U2 can be obtained by analogy.

(8) $$\begin{array}{l} {V_{dq}}^{+}=\left[\begin{array}{c} {V_{d}}^{+}\\ {V_{q}}^{+} \end{array}\right]=V^{+}\left[\begin{array}{c} \cos\varphi^{+}\\ \sin\varphi^{+} \end{array}\right]+V^{-}\cos\varphi^{-}\left[\begin{array}{c} \cos(2\omega t)\\ -\sin(2\omega t) \end{array}\right]+V^{-}\sin\varphi^{-}\left[\begin{array}{c} \sin(2\omega t)\\ \cos(2\omega t) \end{array}\right]\\ {V_{dq}}^{-}=\left[\begin{array}{c} {V_{d}}^{-}\\ {V_{q}}^{-} \end{array}\right]=V^{-}\left[\begin{array}{c} \cos\varphi^{-}\\ \sin\varphi^{-} \end{array}\right]+V^{+}\cos\varphi^{+}\left[\begin{array}{c} \cos(2\omega t)\\ \sin(2\omega t) \end{array}\right]+V^{+}\sin\varphi^{+}\left[\begin{array}{c} -\sin(2\omega t)\\ \cos(2\omega t) \end{array}\right] \end{array}$$

It can be observed from Equation (8) that the coupled components on the positive sequence and negative sequence coordinate axes rotate at twice the angular velocity of the rotor, so four low-pass filters can be introduced to filter them out, as shown by LPF1~LPF4 in Figure 5. The cut-off frequencies of the four low-pass filters are set to $1/\sqrt{2}$ times the frequency of the resolver rotor to ensure faster system response and less system overshoot.

In this software phase-locked loop scheme, the phase-locked algorithm can be smoothly switched between DDSRF-PLL and SRF-PLL by designing the weight coefficient. For example, in order to solve the problem of low bandwidth in the low-speed area and static state, a switch to the SRF-PLL state will achieve a faster response. In the medium and high speed area, a switch to the DDSRF-PLL state will result in a strong anti-interference ability.

## 5. Experimental Results and Analysis

The 200 kW AVL rack adopts the new generation of bench control system PUMA 2.0, uses the most advanced computer system, and has powerful functions, such as big data, which provides a strong guarantee for the test and data processing of the single electronic control or multi-in-one product control system. In addition, the system can perform real-time monitoring and protection of system parameters to ensure the safety of system operation. The rack is also equipped with the latest generation of battery simulators and power analyzers to ensure the efficiency and accuracy of the system in comprehensive testing and verification. In terms of system functions, it has a road load simulation function. By configuring vehicle parameters, it simulates the running state of electronically controlled products in the vehicle, thereby, simplifying the test process, improving test efficiency and reducing test costs. In terms of system parameters, the maximum speed of the system can reach 12,000 RPM, the maximum power is 200 kW, and the system has high speed control accuracy.

The parameters of the AVL rack system are as follows: rated power of 200 kW, rated torque of 450 N/m, rated speed of 4770 RPM, maximum speed of 12,000 RPM; constant power speed of 3858–1200 RPM.

### 5.1. Sine and Cosine Signal Demodulation Test

**Figure 8:**
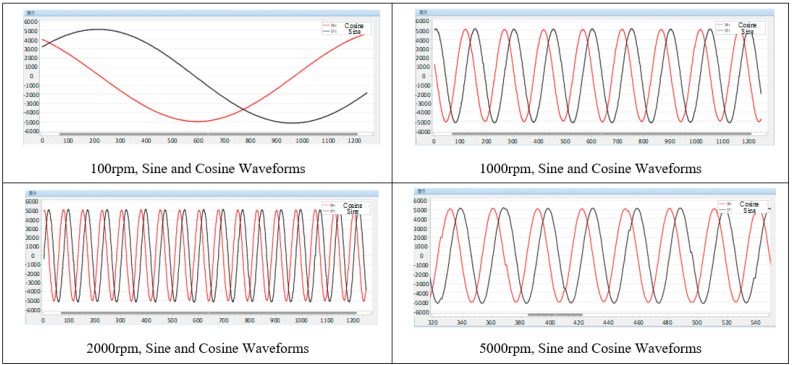
Demodulated sine and cosine waveforms at each rotational speed.

When the motor speed is 100 rpm, 1000 rpm, 2000 rpm, and 5000 rpm, the demodulated sine and cosine signals are captured in the PWM interrupt. As shown in Figure 8, it can be observed that the demodulated sine and cosine signals are relatively smooth, which verifies the effectiveness of the above demodulation scheme.

Among them, at 5000 rpm speed, it can be observed that some data points in the waveform remain unchanged from the last sampling value, which is caused by the execution frequency of the PWM interrupt being higher than the execution frequency of the sine and cosine signal demodulation function. This only affects the sampling observation of the signal and has no effect on the software decoding process.

### 5.2. The Use of the Software PLL Performance Test When the Angle Suddenly Changes

**Figure 9:**
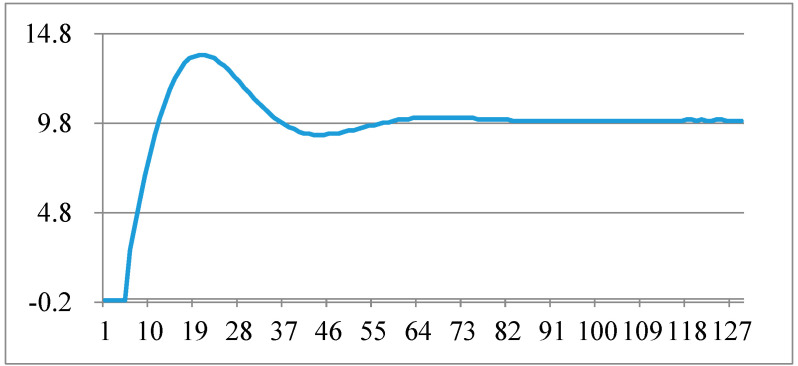
Phase-locked angle test when the angle suddenly changes by 10 degrees.

**Figure 10:**
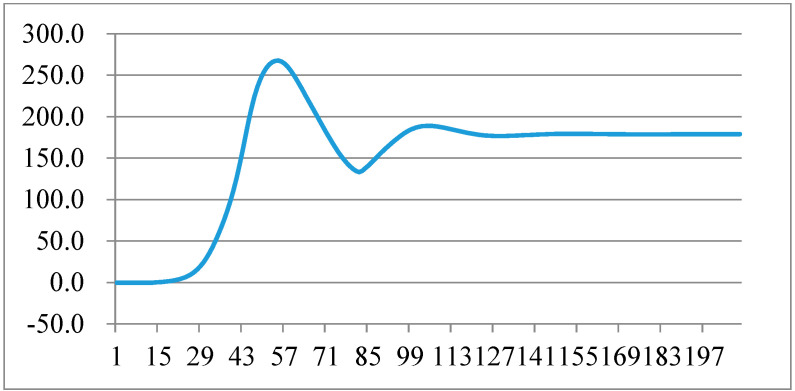
Phase-locked angle test when the angle suddenly changes by 179 degrees.

In order to verify the performance of the software phase-locked loop, the input sine and cosine signals are processed for step change, and the change in the phase-locked output angle of the DDSRF-PLL is observed. In the following figure, the frequency of data capture is 5 kHz, the unit of ordinate is degree, and the abscissa is the number of points of the captured data. The waveform of the sudden change in angle by 10 degrees is shown in Figure 9, and the adjustment time is about 11 ms. The waveform of the sudden change in angle by 179 degrees is shown in Figure 10, and the adjustment time is about 21 ms.

### 5.3. Comparison Test of Software Decoding and Hardware Decoding

**Figure 11:**
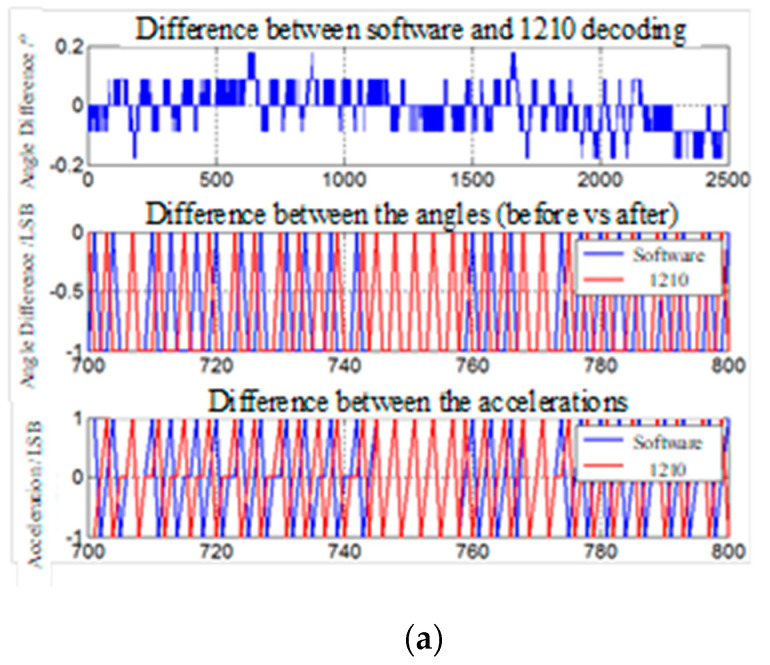

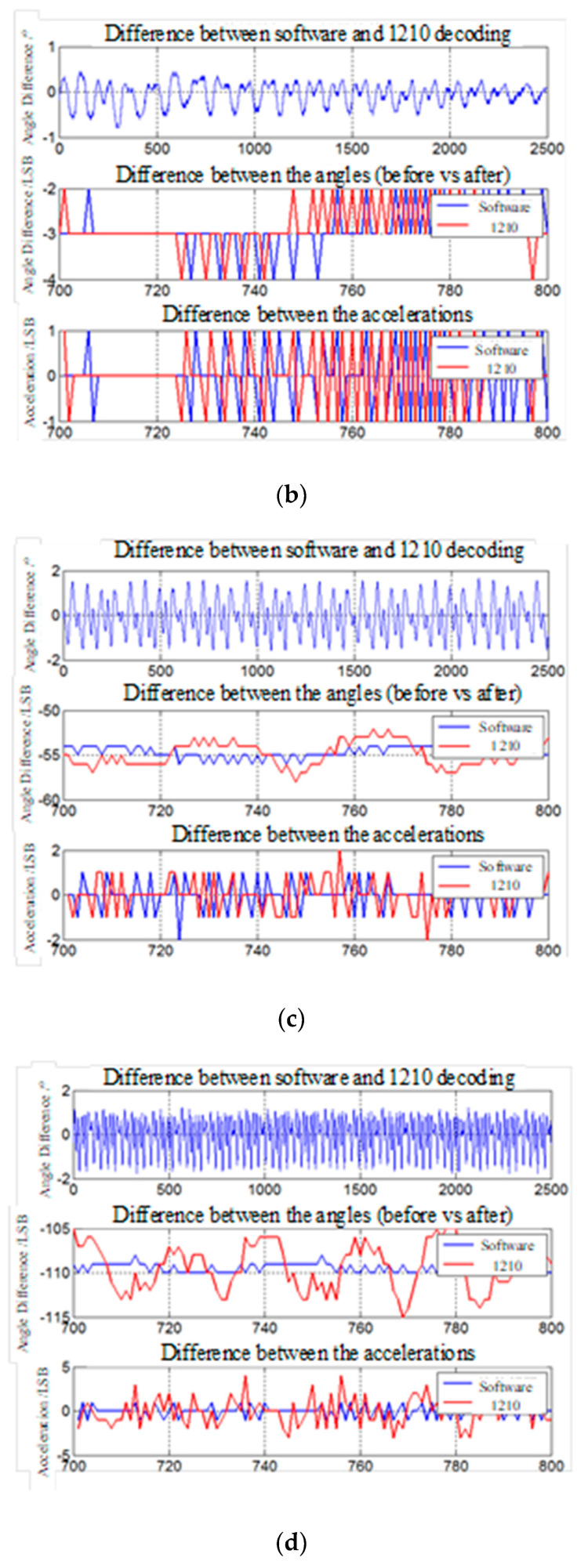

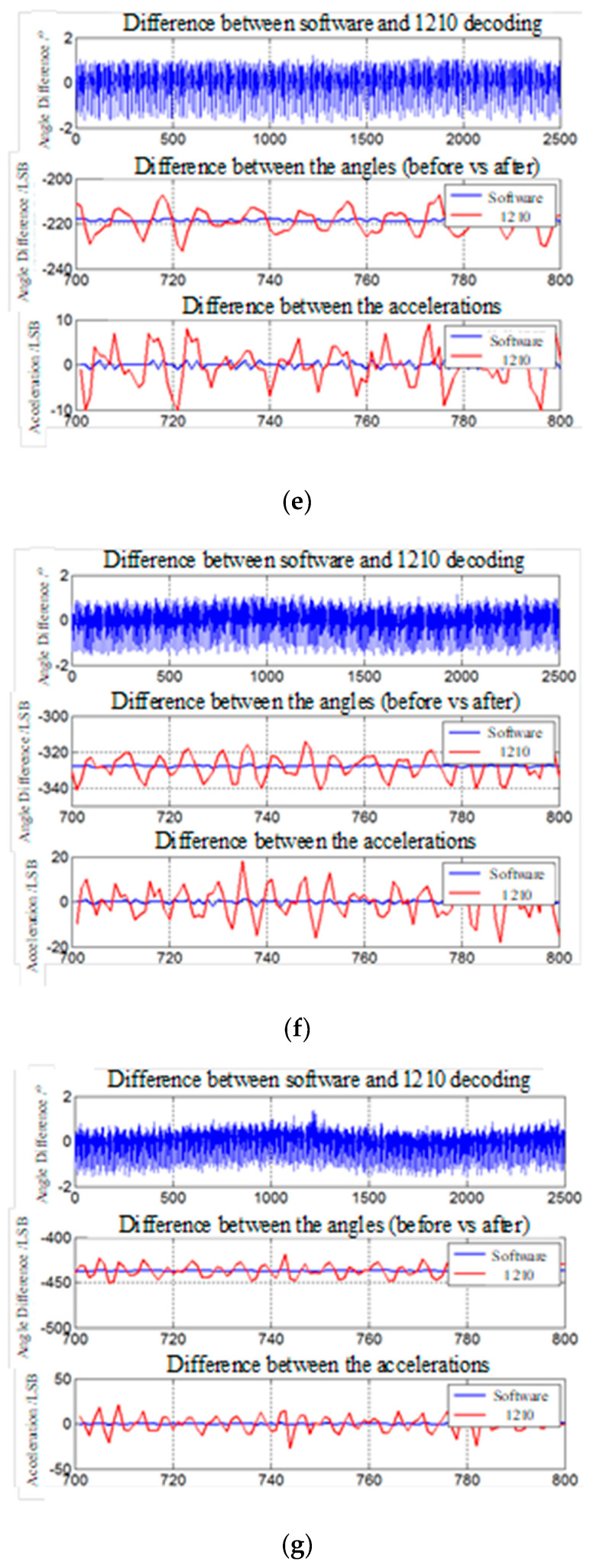

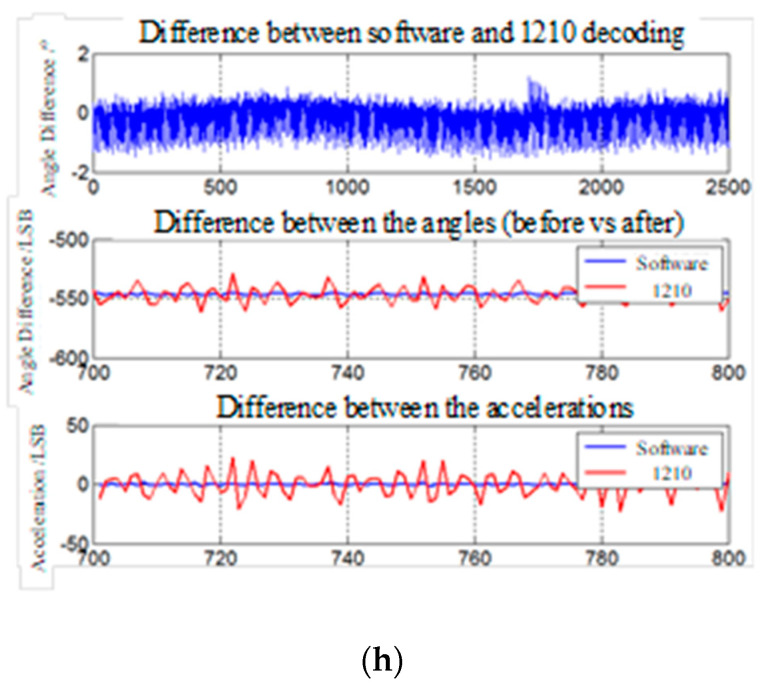
Comparing waveforms of software decoding angle and hardware decoding angle at different speeds: (**a**) 10 rpm experimental waveforms; (**b**) 50 rpm experimental waveforms; (**c**) 1000 rpm experimental waveforms; (**d**) 2000 rpm experimental waveforms; (**e**) 4000 rpm experimental waveforms; (**f**) 6000 rpm experimental waveforms; (**g**) 8000 rpm experimental waveforms; (**h**) 10,000 rpm experimental waveforms.

As shown in Figure 11, the experimental waveforms at different rotational speeds are divided into three, and the top waveform is the difference between the software decoding angle and the 1210 decoding angle, which is converted to degree. The middle waveform is the difference between the current angle and the previous angle, the precision is set to 12 bits, and the unit is 1 LSB. The bottom waveform is the difference between the current speed and the previous speed, that is, the acceleration waveform, and the unit is 1 LSB.

As we can be observe from Figure 11, when the speed is 10 rpm, the angular amplitude of the two methods is within 0.2 degrees, and the speed and acceleration can be tracked similarly. When the speed is 50 rpm, the angle amplitude remains at 0.5 degrees, and the speed changes linearly and similarly. When the speed exceeds 1000 rpm, the amplitude of the angle difference remains within 1 degree. The two methods are always close and accurate for speed tracking, and the oscillation amplitude decoded by software is smaller for acceleration tracking.

According to the experimental results in Figure 11, it can be observed that the proposed scheme has a good filtering effect, and the oscillation amplitude of the velocity waveform and acceleration waveform under the software decoding in the medium and high speed region is much smaller. On the whole, the error between the angle obtained by software decoding and the angle obtained by hardware decoding is very small, and one of them can be used as the angle feedback for motor control, and the other is used as a redundant angle to monitor the state of the system. When the angle error of the two exceeds the set threshold, it can be known that the decoder or the position detection loop is faulty, and the system will alarm and stop to ensure the safety of the system.

## 6. Conclusions

In the field of motor control, especially in the field of electric vehicles, people pay more and more attention to the safety of the control system, and the functional safety of the system has become an important indicator. A single method of motor rotor position acquisition cannot meet the requirements of system functional safety. Therefore, this paper integrates hardware decoding and software decoding in the motor control system at the same time. The resolver decoding chip is used to generate the excitation voltage, and the decoding chip and software decoding run at the same time. The angle data are obtained by two decoding methods, one is used for the current motor control of the system, and the other is used as a redundant angle decoding algorithm to improve the functional safety of the entire motor control system. Finally, the effectiveness of the proposed simultaneous operation of hardware decoding and software decoding is verified by experiments.

## Data Availability

Not applicable.
